# Blue light excited retinal intercepts cellular signaling

**DOI:** 10.1038/s41598-018-28254-8

**Published:** 2018-07-05

**Authors:** Kasun Ratnayake, John L. Payton, O. Harshana Lakmal, Ajith Karunarathne

**Affiliations:** Department of Chemistry and Biochemistry, The University of Toledo, Toledo, OH 43606 USA

## Abstract

Photoreceptor chromophore, 11-cis retinal (11CR) and the photoproduct, all-trans retinal (ATR), are present in the retina at higher concentrations and interact with the visual cells. Non-visual cells in the body are also exposed to retinal that enters the circulation. Although the cornea and the lens of the eye are transparent to the blue light region where retinal can absorb and undergo excitation, the reported phototoxicity in the eye has been assigned to lipophilic non-degradable materials known as lipofuscins, which also includes retinal condensation products. The possibility of blue light excited retinal interacting with cells; intercepting signaling in the presence or absence of light has not been explored. Using live cell imaging and optogenetic signaling control, we uncovered that blue light-excited ATR and 11CR irreversibly change/distort plasma membrane (PM) bound phospholipid; phosphatidylinositol 4,5 bisphosphate (PIP2) and disrupt its function. This distortion in PIP2 was independent of visual or non-visual G-protein coupled receptor activation. The change in PIP2 was followed by an increase in the cytosolic calcium, excessive cell shape change, and cell death. Blue light alone or retinal alone did not perturb PIP2 or elicit cytosolic calcium increase. Our data also suggest that photoexcited retinal-induced PIP2 distortion and subsequent oxidative damage incur in the core of the PM. These findings suggest that retinal exerts light sensitivity to both photoreceptor and non-photoreceptor cells, and intercepts crucial signaling events, altering the cellular fate.

## Introduction

Light activatable G-protein coupled receptors (GPCRs), also known as opsins, harvest light through their covalently bound chromophore 11-*cis* retinal (11CR), an aldehyde derivative of vitamin A^1,2^. Once an opsin is activated, all *trans* retinal (ATR) is released, and converted back to 11CR within the retinal pigment epithelium (RPE) by a multi-component ATR clearance mechanism composed of retinaldehyde dehydrogenase, RPE65, lecithin retinol acyltransferase and ATP-binding cassette transporter A4^3^. Dysfunctions in 11CR regeneration process result in ATR accumulation in the retina^3–5^. ATR mediated cytotoxicity and associated pathological conditions have been reported^5–8^. Studies in mice have demonstrated that, ATR accumulation and photodegradation leads to diseases such as age related macular degeneration (AMD), Stargardt disease, acute light-induced retinopathy, retinitis pigmentosa and night blindness^5^. Although retinal undergoes degradation upon exposure to light, neither photodegradation pathways leading to specific photoproducts formation nor plausible non-visual signaling of these photoproducts are sufficiently understood^6^. Several mechanisms of retinal induced toxicity in photoreceptor cells have been proposed^5,9,10^. In the retina, higher ATR concentrations have been linked to cytotoxicity due to their ability to form oxidized condensation products known as lipofuscins^10–14^. Lipofuscins include ATR dimers/adducts, N-retinylidene-N-retinylethanolamine (A2E), N-retinylidene and N-retinylethanolamine. While lipofuscins can produce reactive oxygen species (ROS) with a low quantum yield^15^, photooxidation of ATR in photoreceptor cells is linked to NADPH oxidase activation, ROS generation and calcium mobilization, inducing cytotoxicity and apoptosis^3,5,8,9,16^. Additionally, photodegraded ATR is linked to cytotoxicity observed in ARPE-19 retinal pigment epithelium cells^6^. Since cytosolic calcium is increased by GPCR activation^17–20^, it has been suggested that ATR induces phototoxicity by interacting with a ligand binding Gq-coupled GPCR, activating phosphatidylinositol pathway through an unknown mechanism^5^. Collectively, if visible light photosensitizes free retinal in cells, what subcellular location chemically supports retinal photochemistry, and what subsequent signaling perturbations photoexcited retinal elicits in living cells are not known.

The present study examines mechanisms of photoexcited retinal intercepting signaling networks in living cells, especially perturbing phospholipid, phosphatidylinositol 4,5 bisphosphate (PIP2) signaling. Results show that photoexcited retinal mediated PIP2 signaling perturbation is independent of GPCR activation. Since PIP2 has been identified as a crucial regulator of cellular functions including cytoskeleton remodeling, cell migration, endocytosis, cell motility and cell shape^21,22^, perturbation of PIP2 signaling by photoexcited retinal could significantly affect cellular physiology. Overall, our data elaborate molecular underpinnings of retinal associated cytotoxicity and its physiological consequences.

## Results

### Retinal absorbs blue light and induces translocation of PIP2 sensor to the cytosol

**Figure 1:**
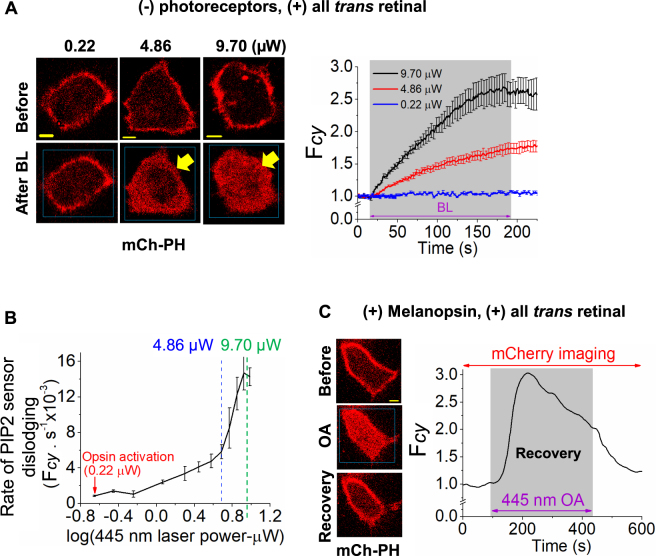
Comparison of photoreceptor dependent PIP2 hydrolysis vs photoreceptor independent PIP2 sensor translocation by photoexcited retinal. (**A**) Images of HeLa cells incubated with 50 µM ATR (retinal) expressing PIP2 sensor (mCherry-PH). Both images and the plot of *F*_*cy*_ vs time show that cells exposed to 0.22 µW 445 nm blue light did not respond, while cells exposed to 4.86 µW and 9.70 µW blue light exhibited mCherry-PH translocation to cytosol (mean ± S.E.M.). (**B**) The plot of initial PIP2 sensor dislodging rate vs laser power of 445 nm blue light. (mean ± S.E.M., n = 6 cells in each experiments). (**C**) Images of HeLa cells expressing Gq-coupled Melanopsin and mCherry-PH. Cells were incubated with ATR (50 µM) for 5 minutes. A significant PIP2 hydrolysis was observed upon optical activation (OA = blue box) of melanopsin using short pulses of blue light (0.22 µW of 445 nm). Recovery of PIP2 sensor to the PM was observed even the blue light exposure is continued. The plot shows the dynamics of PIP2 sensor translocation in cytosol. Mean and S.E.M. are from 3 < independent experiments. (blue light (BL) = blue box). Scale = 5 µm.

The objective was to examine whether retinal or blue light excited retinal, independent of photoreceptors, elicits PIP2 hydrolysis and inositol (1,4,5) triphosphate (IP3) generation in cells, because calcium and its regulatory pathways are suggested as key players of cytotoxicity in the retina^5,23^. We employed HeLa cells as the major cell line here to eliminate potential response contamination due to retinal and blue light activating endogenous photoreceptors in cells derived from retina. Upon retinal addition to HeLa cells expressing PIP2 sensor (mCherry-PH), cells did not show any change in sensor distribution (Fig. 1A, left). These cells were exposed to blue light at every one-second interval for 10 minutes. Starting blue light intensity was set to 0.22 µW, since this power is sufficient to activate retinal bound to photoreceptors. Cells were imaged for mCherry while gradually increasing blue light intensity. Intensities at 1 μW and above, cells exhibited mCherry-PH translocation to cytosol (Fig. 1A,B, Movie-S1). However, at these intensities, photobleaching of fluorescence proteins was not observed. Interestingly, the translocated mCherry-PH did not recover, even after termination of blue light exposure, suggesting the likelihood of irreversible photochemical perturbation of PIP2 by blue light excited retinal.

Response signatures of mCherry-PH translocation induced by blue light (4.86 μW) excited retinal (BLE-retinal) were compared with PIP2 hydrolysis elicited by melanopsin-Retinal Schiff Base (RSB), to examine if both processes trigger similar chemical changes to PIP2. HeLa cells expressing melanopsin and mCherry-PH were incubated with ATR (50 µM) for 5 minutes in dark to allow RSB formation. Cells exhibited PIP2 hydrolysis upon exposure to low intensity blue light (0.22 µW), as indicated by the translocation of mCherry-PH from PM to cytosol (Fig. 1C, Movie-S2). The observed PIP2 hydrolysis was transient and recovered within 2–3 minutes. Gq-coupled GPCR activation induced-PIP2 hydrolysis recovers over time due to signaling adaptation^24,25^. Similar to melanopsin activation, addition of carbachol to HeLa cells expressing M3R and PIP2 sensor exhibited PIP2 hydrolysis. A similar recovery of PIP2 was also observed (Fig. S1, Movie-S3).

**Figure 2:**
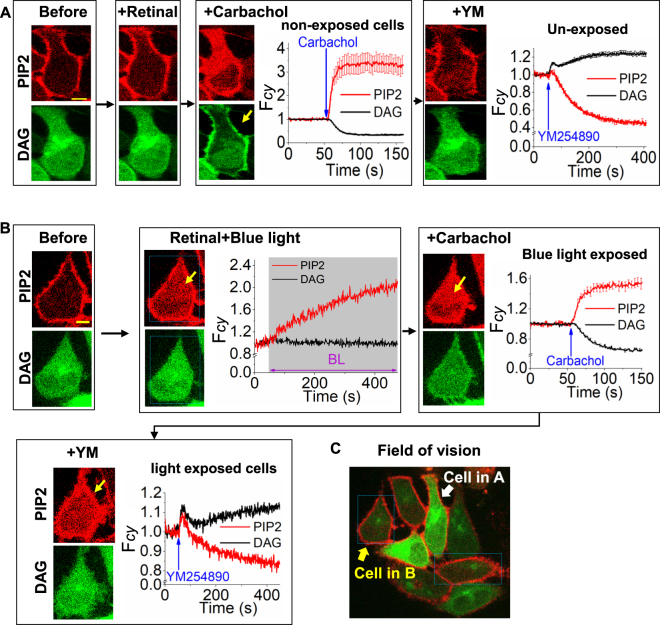
Comparison of M3-muscarnic receptor mediated PIP2 hydrolysis vs blue light excited retinal (BLE-retinal) induced PIP2 sensor translocation. Images of HeLa cells expressing M3-muscarinic receptor, mCherry-PH (PIP2 sensor), DBD-YFP (DAG sensor). (**A**) Without blue light exposure, retinal addition does not change PIP2 or DAG sensor distribution (left), while the addition of carbachol resulted in PIP2 hydrolysis and DAG formation (middle). The addition of Gq inhibitor, YM254890 (1 µM) to cells resulted in reverse translocation of PIP2 and DAG sensors. (mean ± S.E.M., n = 6 cells) (**B**) When cells were exposed to blue light (4.86 µW of 445 nm) in the presence of retinal, they only showed PIP2 sensor translocation while no change in DAG sensor was observed (left). The addition of carbachol to these cells exhibited an additional PIP2 sensor translocation to the cytosol with a mild DAG sensor translocation to the PM (left) (mean ± S.E.M., n = 4 cells). Interestingly, addition of YM254890 only reversed both PIP2 and DBD responses elicited by carbachol. (**C**) The field of vision of cells shown in A and B. Mean and S.E.M. are from 3 < independent experiments. (blue light (BL) = blue box). Scale = 5 µm.

During PIP2 hydrolysis, IP3 moiety of PIP2 dissociates from PM anchored diacylglycerol (DAG) domain. The PH domain of PLCδ1 in the PH sensor moves to cytosol with IP3 upon PIP2 hydrolysis^26^. The irreversible translocation of mCherry-PH by BLE-retinal was further characterized to determine if this is due to PIP2 hydrolysis or a photochemical perturbation to PIP2, either by breaking the molecule or preventing PIP2 sensor binding. To examine whether BLE-retinal induces mCherry-PH translocation due to PIP2 hydrolysis, leaving DAG on the PM, DAG generation was imaged using cytosolic DAG sensor, YFP-DBD, that translocates to PM. Cells incubated with ATR failed to exhibit a detectable mCherry-PH translocation (Fig. 2A). When a selected cell in the field of vision (Fig. 2C, yellow arrow) was exposed BLE-retinal for 400 seconds, a robust mCherry-PH translocation only in blue light exposed cell was observed (Fig. 2B). However, the DAG sensor did not translocate to PM (Fig. 2B), indicating that, exposure to BLE-retinal does not leave DAG at PM. We also comparatively examined Gq-coupled M3-muscarinic receptor (M3R) activation in the same HeLa cells transiently expressing M3R. When cells were exposed to 10 µM carbachol, both PIP2 hydrolysis and DAG formation were observed (Fig. 2A). When this cell was exposed to YM254890 (1 µM), which inhibits Gαq heterotrimer activation, both PIP2 and DAG sensors reversed to their basal pre-activation status, suggesting PIP2 recovery (Fig. 2A). Interestingly, the cell exposed to BLE-retinal did not show such a recovery upon addition of YM254890 (Fig. 2B). This further confirms that BLE-retinal induced PIP2 distortion in cells is irreversible.

### Characteristics of retinal photoexcitation in cellular environment

**Figure 3:**
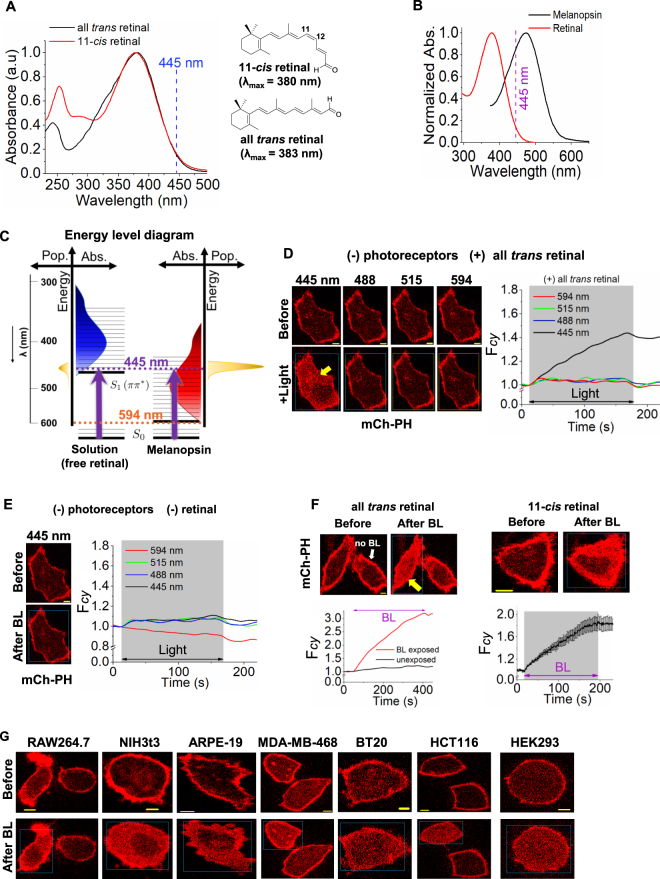
Energy and wavelength requirement for PIP2 solubilization by photoexcited retinal. (**A**) UV-VIS absorption spectra for both 11-cis retinal (11CR) and all *trans* retinal (ATR). Note that 445 nm blue light spectrally overlaps with both absorption spectra. (**B**) The absorption spectra of melanopsin and retinal (left), (ε_ATR_ = 44180.0 M^−1^cm^−1^). (**C**) The energy level diagram and the population (pop.) of energy levels of free retinal (blue) and melanopsin (red) according to their respective absorption maxima (right). Note that blue light (445 nm) can highly populate melanopsin compared to that of free retinal. (**D**–**F**) Images of HeLa cells expressing PIP2 sensor (mCherry-PH). (**D**) Cells were incubated with ATR (50 µM) for 5 minutes. A substantial PIP2 sensor translocation was observed upon exposing cells to short pulses of blue light (4.86 µW of 445 nm). The plot shows the dynamics of PIP2 sensor translocation to cytosol. (**E**) In the absence of retinal, cells did not show a detectable PIP2 sensor translocation when exposed to blue light or other wavelengths. (**F**) Both blue light excited ATR (50 µM) and 11CR (50 µM) exhibited a permanent accumulation of PIP2 sensor cytosol. Compared to exposed cell (yellow arrow), control cell without blue light (BL) exposure (white arrow) did not show any detectable PIP2 response. The plots show the dynamics of PIP2 sensor translocation in cells shown in F (mean ± S.E.M., n = 6 cells). (**G**) All *trans* retinal and blue light induce PIP2 sensor translocation in cells with distinct origins. Images of RAW264.7, NIH3t3, ARPE-19, MDA-MB-468, BT-20, HCT116 and HEK293 cells expressing mCherry-PH (PIP2 sensor). ATR (50 µM) was incubated in cells for 5 minutes followed by continuous exposure of blue light for 5 minutes. Blue light exposure induced PIP2 sensor translocation from PM in all the cell types tested while cells that were not exposed to blue light did not respond. Mean and S.E.M. are from 3 < independent experiments. (blue light (BL) = blue box). Scale = 5 µm.

Compared to the light power required for opsin activation (~0.2 μW), a higher power of blue light (>1 μW) was required for free retinal to induce PIP2 distortion. This can be partly due to the bathochromic shift in the absorption spectrum of melanopsin-retinal Schiff base (m-RSB) with a λ_max_ = 478 nm, compared to the λ_max_ = 383 nm of free retinal (Fig. 3A,B). This allows m-RSB to have a greater spectral overlap with blue light (445 nm) that drives a larger population of molecules to the excited S_1_(π* ← π) state, compared to free retinal (Fig. 3C). Since quantum yield of m-RSB isomerization is estimated to be at near unity^27–29^, all absorbed photons result in GPCR activation. Additionally, the lower power requirement for melanopsin activation induced PIP2 hydrolysis is due to the enhanced photosensitivity of m-RSB and PIP2 hydrolysis being an enzymatic process while BLE-excited retinal induced PIP2 distortion is likely to be a non-enzymatic and stoichiometric reaction.

To examine the wavelength dependency of BLE-retinal induced PIP2 distortion, HeLa cells expressing mCherry-PH were exposed to 445 nm, 488 nm, 515 nm and 594 nm wavelengths of light at 4.86 µW respectively, in the presence or absence of retinal. Cells exposed to 488, 515 and 594 nm light, in the presence of retinal did not show a detectable signal (Fig. 3D). Control cells also did not exhibit PIP2 distortion with blue light in the absence of retinal (Figs 3E and S3). Retinal addition alone (without blue light) also showed no PIP2 distortion (Fig. 3F). Only the selected cell in the field of vision exposed to blue light exhibited PIP2 distortion (Fig. 3F, yellow arrow, Movie-S4). Similar to ATR, 11CR also induces PIP2 distortion when exposed to blue light (Fig. 3F). Unless specified, all the experiments henceforward employed blue light with the power of 4.86 µW to excite retinal. At this light power, photobleaching of fluorescent biosensors was not observed. All the cell types examined including RAW264.7 (mouse macrophage), ARPE-19, MDA-MB-468 (triple negative breast cancer), BT20 (triple negative breast cancer), HCT116 (colon cancer), NIH3t3 (mouse embryonic fibroblast), and HEK293 (human embryonic kidney) exhibited mCherry-PH translocation upon exposure to BLE-Retinal (Fig. 3G) which indicates a universal mechanism operated by BLE-retinal.

**Figure 4:**
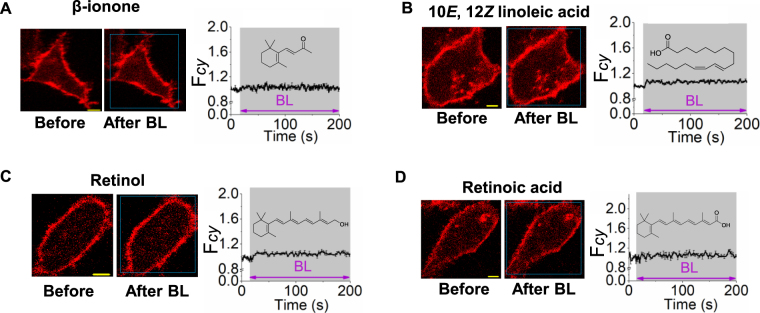
Retinal-like molecules exhibited no effect on PIP2 upon blue light exposure. Images of HeLa cells expressing PIP2 sensor (mCherry-PH) on PM. In all of the experiments conducted, cells were exposed to blue light (4.86 µW of 445 nm) which is indicated by the white box. Cells were incubated with (**A**) β-ionone (50 µM), (**B**) 10*E*, 12*Z* linoleic acid (50 µM), (**C**) Retinol (50 µM) and (**D**) Retinoic acid (50 µM), for 10 minutes followed by continuous exposure of short pulses of blue light for 200 s. In all experiments, cells did not show a detectable PIP2 sensor translocation. Plots show the dynamics of PIP2 sensor translocation in cells shown in **A**–**E**. (mean ± S.E.M., n = 5–10 cells). Mean and S.E.M. are from 3 < independent experiments. (blue light (BL) = blue box). Scale = 5 µm.

To decipher molecular features of retinal for its blue light-induced cellular effects, compounds with analogous functional groups such as retinol, retinoic acid, β-ionone (β-ionone ring of retinal) and conjugated linoleic acid (CLA- mimicking the extended π conjugated system) were examined as follows. HeLa cells expressing mCherry-PH were incubated for 10 minutes with the retinal-like molecules at 50 μM concentrations (Fig. 4). Interestingly, none of these compounds were able to induce PIP2 distortion upon exposure to blue light (Fig. 4A–D). Examination of absorption spectra of these screened molecules show that they do not have sufficient spectral overlaps with the blue light (Fig. S3). However, both retinol and retinoic acid exhibited mCherry-PH translocation when exposed to 365 nm ultra violet light (Fig. S4).

### BLE-retinal induced PIP2 distortion is independent of GPCR-G protein activation

**Figure 5:**
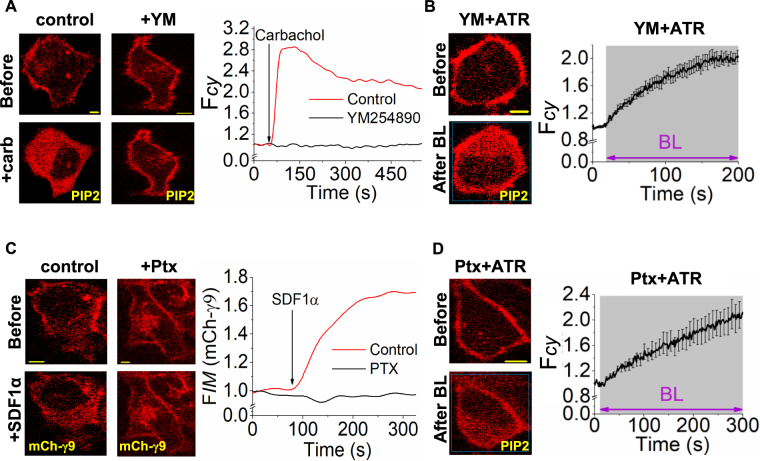
Photoexcited retinal induced PIP2 translocation is not due to GPCR pathway activation. (**A**) HeLa cells expressing M3 receptor and mCherry-PH, carbachol (10 µM) was added to activate M3R in the presence (left) and absence (middle) of Gq inhibitor (YM254890, 1 µM, 5 min). Only control cells (no YM254890), showed PIP2 hydrolysis (left). (**B**) Even in the presence of Gq inhibitor, blue light excited retinal induced PIP2 sensor translocation (middle). The plots show the dynamics of PIP2 sensor translocation in the cells shown in A and B. (**C**) To HeLa cells expressing CXCR4-GFP, mCh-γ9, SDF1α (100 ng/mL) was added to activate CXCR4 in the presence (left) and absence (middle) of Gαi inhibitor (pertussis toxin = Ptx, 50 ng/mL, overnight incubation). Only control cells with no added Ptx exhibited mCh-γ9 translocation from PM to IMs (left). (**D**) Exposure to photoexcited retinal induced PIP2 sensor translocation in cells treated with Ptx. The plots show the dynamics of mCh-γ9 and PIP2 sensor translocation. In all the experiments conducted, cells were exposed to 4.86 µW of 445 nm blue light. (mean ± S.E.M., n = 5–10 cells). Mean and S.E.M. are from 3 < independent experiments. (blue light (BL) = blue box). Scale = 5 µm.

Retinal and blue light mediated activation of Gq-coupled GPCRs are suggested to trigger signaling and cytotoxicity in ARPE-19 photoreceptor cells^5^. This study measured degeneration of cells in the mouse retina, however the underlying mechanism was unclear. Upon GPCR activation, both Gαq-GTP and Gβγ can induce PIP2 hydrolysis since they activate PLCβ^30^. To examine whether BLE-retinal activates GPCR-G proteins pathways, PIP2 distribution was studied in the presence of Gαq inhibitor-YM254890 (1 µM) and Gi-pathway inhibitor-pertussis toxin (PTX) (50 ng/mL) respectively (Fig. 5A–D). Previously, we showed that pertussis toxin inhibits Gβγ mediated PIP2 hydrolysis since inhibition of Gi-pathway activation prevents generation of free Gβγ^31^. After inhibitor treatment, cells incubated with retinal were exposed to blue light for 2–3 minutes while imaging the PIP2 sensor response. Both inhibitors failed to inhibit BLE-retinal induced PIP2 distortion (Fig. 5B,D). Additionally, HeLa cells were treated with both Gαq and Gβγ inhibitors simultaneously to rule out the possibility of activation of PLCβ by BLE-retinal. Regardless of the collective inhibition of Gβγ and Gαq, BLE-retinal induced PIP2 disruption in cells (Fig. S5). This indicates that BLE-retinal induced responses are not due to the GPCR-G protein pathway activation. Control experiments show YM254890 and PTX inhibit Gq-pathway induced PIP2 hydrolysis and Gi-pathway induced Gγ9 translocation, respectively (Fig. 5A,C)^32^. In addition, retina-derived ARPE-19 cells exhibited PIP2 disruption upon BLE-retinal in the presence of Gαq, Gβγ and Gi pathway inhibitors (Fig. S6). Contrary to the previous reports^7^, these experiments and data confirm that free retinal with or without light does not activate GPCR pathways.

### PIP2 distortion by BLE-retinal is associated with an increase in intracellular calcium, [Ca^2+^]_i_

**Figure 6:**
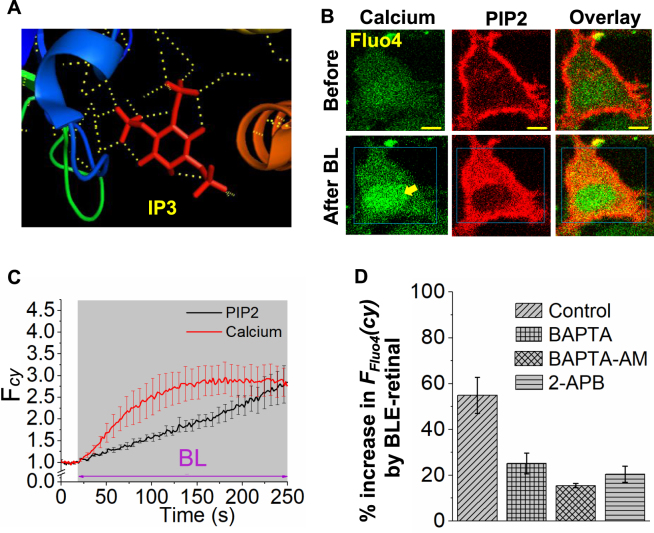
All trans retinal and blue light induced signaling in cells. (**A)** Crystal structure of the IP3 receptor bound with IP3 with H-bonding interactions (PDB code:1N4K), suggesting the PIP2 can have the majority of interactions exhibited by IP3. (**B)** HeLa cells expressing PIP2 sensor (mCherry-PH) and incubated with calcium sensor Fluo4. Fluo4 stained cells were incubated with ATR (50 µM) for 5 minutes, followed by exposure of blue light (4.86 µW of 445 nm) for 3 minutes. Here the whole cell was exposed to blue light. A substantial increase in cytosolic calcium is observed. (**C**) Dynamics of calcium responses and PIP2 translocation in the cells shown in B (mean ± S.E.M., n = 5 cells). (**D**) Calcium responses in control and calcium modulator-incubated (using BAPTA-AM and 2-APB) HeLa cells in regular and extracellular calcium free (using BAPTA) buffers. Here, cells were pre-incubated Fluo4 were incubated with 2-APB (5 µM for 15 min), BAPTA-AM (10 µM for 30 min), or BAPTA (5 µM for 5 min) in calcium free HBSS buffer. The cells were then incubated with ATR (50 µM) for 5 minutes, followed by continuous exposure of blue light for 5 minutes. The bar chart shows the changes in calcium sensor fluorescence in the cytosol before and after blue light exposure on cells for all the above mentioned experiments (mean ± S.E.M., n = 5–15 cells). Mean and S.E.M. are from 3 < independent experiments. (blue light (BL) = blue box). Scale = 5 µm.

According to the crystal structure of IP3 receptor^33^, IP3 molecule forms H-bonds with the putative interacting domain (Fig. 6A, PDB code:1N4K). To examine whether distorted PIP2, that may be entering cytosol could also increase cytosolic calcium, HeLa cells expressing mCherry-PH were incubated with cell permeable calcium sensor fluo4-AM and exposed to BLE-retinal. Cells exhibited a robust upsurge of calcium and a synchronized mCherry-PH translocation response was observed (Fig. 6B,C, Movie-S5). Unlike Gq-coupled GPCR induced calcium response, which recovers over time (Fig. S7A), BLE-retinal induced response did not exhibit signaling adaptation (Fig. 6C). To examine the source of calcium mobilized by BLE-retinal, cellular calcium modulators were deployed as follows and percent change in fluo-4 fluorescence in the cell upon exposure to BLE-retinal was calculated with respect to fluo-4 fluorescence observed prior to blue light exposure. Upon BLE-retinal exposure, cells without a calcium modulator was considered as the control and exhibited a 55 ± 8% increase in fluo4 fluorescence compared to basal fluorescence. Cells incubated in extracellular calcium free medium (using BAPTA) and with intracellular calcium chelator, BAPTA-AM, induced only 25 ± 5% and ~16 ± 1% increases respectively, (Fig. 6D). Treating cells with IP3 receptor inhibitor, 2-aminoethoxydiphenyl borate (2-APB) resulted in ~21 ± 4% increase (Fig. 6D). These data indicate possible interactions of the IP3 domain of distorted PIP2 with IP3 receptors^33^. Calcium modulators above were validated as follows (Fig. S7). Cells incubated with BAPTA-AM showed only PIP2 hydrolysis but no calcium response upon M3-muscarinic receptor activation (Fig. S7B), while 2-APB treated cells showed a transient PIP2 hydrolysis with no calcium response (Fig. S7C). Compared to BLE-retinal induced calcium responses, M3R activation induced responses were rapid and transient. These results also show the ability of BLE-retinal to induce downstream signaling, independent of GPCR activation. Collectively, these data suggest that BLE-retinal induced distortion of PIP2 not only induces mobilization of stored calcium, but also stimulate influx of extracellular calcium.

### Blue light-excited retinal induces cytotoxicity

**Figure 7:**
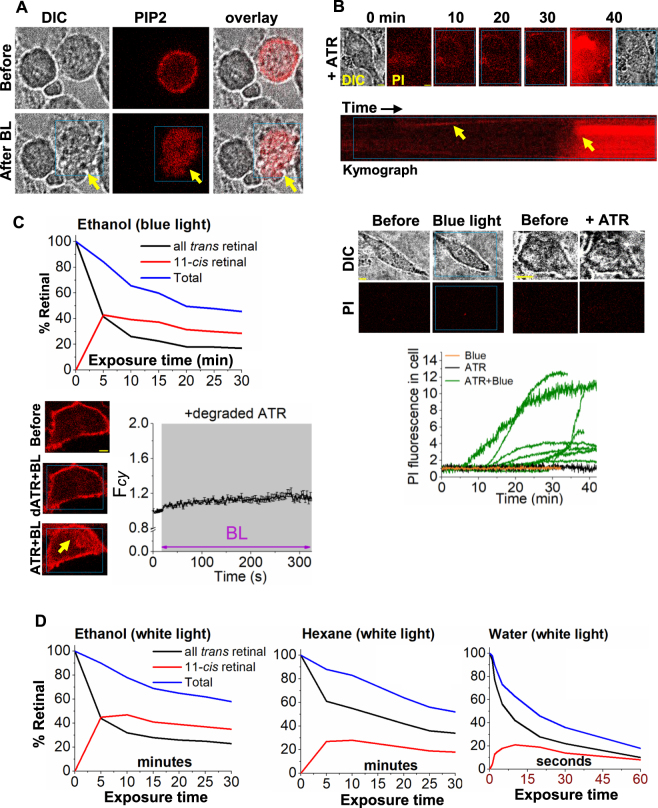
Photoexcited retinal induced cytotoxicity. (**A**) Fluorescence and DIC images of HeLa cells treated with ATR (50 µM) followed by blue light exposure (4.86 µW of 445 nm). Only the middle cell (yellow arrow) expresses PIP2 sensor. Blue light exposed (blue box) cell showed substantial change in cell shape and morphology. The PIP2 sensor also accumulates in cytosol upon blue light exposure. (**B**) HeLa cells were incubated with propidium iodide (PI) with ATR (50 µM) and exposed to blue light (4.86 µW) for 45 minutes. Incorporation of PI in to cells were observed upon light exposure. The control experiments performed with cells exposed to only to blue light or only to ATR, did not show PI incorporation into cells over time. Plot shows the different rates of PI incorporation into cells compared to control experiments. (**C**,**D)** Solvent dependent degradation and isomerization of ATR. (**C**) ATR (20 µL of 50 mM in ethanol) was exposed to blue LED light for 30 minutes. The blue light exposed ATR (injection sample: 1 µL of exposed ATR was diluted in 1 mL of ethanol) was analyzed by HPLC where degradation of ATR is observed by reduction of corresponding ATR peak in chromatogram. *right:* The degraded ATR (dATR) (1 µL) was added to HeLa cells (final volume of imaging buffer = 1 mL) expressing PIP2 sensor and continuously exposed to 445 nm light (4.86 µW). Cells did not show detectable PIP2 translocation upon blue light. Exposure of cells to fresh ATR (50 µM) and blue light (BL) induced PIP2 distortion (mean ± S.E.M., n = 12). (**D**) HPLC analysis of retinal in different solvents after exposing to white light for varying durations. Note that retinal in water degrades in *seconds* while in ethanol and hexane show over 100 times enhanced stability. Improved isomerizations were seen as well. Mean and S.E.M. are from 3 < independent experiments. (blue light (BL) = blue box). Scale = 5 µm.

The possibility of PIP2 distortion and calcium mobilization elicited by BLE-retinal to induce cytotoxicity was examined. Morphology of HeLa cells incubated in retinal containing medium was observed using time-lapse microscopy while only selected cells were exposed to blue light. PIP2 distortion and substantial morphological changes, including extensive bleb formation, were observed only in blue light exposed cells (Fig. 7A, Movie-S6). Unexposed control cells in the same field remained morphologically intact. To identify whether exposure of BLE-retinal induces cell death, HeLa cells were incubated with cell-death marker, propidium iodide (PI) and examined its inclusion, since apoptotic cells fail to exclude PI^34,35^. Cells were supplemented with 100 µL of PI (50 µg/mL) and 50 µM ATR, while selected cells were exposed to blue light. Control cells were either exposed to blue light without retinal or incubated with retinal without blue light exposure. Cells exposed to blue light in the presence of retinal showed gradual incorporation of PI, indicated by the increase in red fluorescence (Fig. 7B). Observed time curves of PI incorporation into cells were heterogenous (Fig. 7B, plot). Cells in both the control experiments did not incorporate PI (Fig. 7B). These results indicate that prolonged exposure of cells to BLE-retinal leads to cell death.

### mCherry-PH sensor translocation by BLE-retinal indicates distortion of the PIP2 molecule

To determine whether translocation of mCherry-PH is a common response exhibited by fluorescence proteins on exposure to BLE-retinal, a membrane anchor with a transmembrane domain (from mouse ICAM5) and a C-terminal mCherry, DenMark-mCherry was used^36^. In addition to DenMark-mCherry, HeLa cells were also expressed with YFP-PH. Upon exposure to BLE-retinal, PM localization of DenMark did not change, while YFP-PH translocated to cytosol (Fig. S8A,C). A saturated lipid anchor bearing glycoprotein, glycosylphosphatidylinositol (GPI) which anchors extracellularly to cells also did not respond to BLE-retinal (Fig. S8B,C). HeLa cells expressing GPI-GFP and NIH3t3 cells expressing GPI-mCherry were used for the experiment. BLE-retinal did not remove GPI anchored proteins from PM. This suggests that specifically PIP2, that contains an unsaturated arachidonoyl chain is susceptible to photochemical reactions elicited by BLE-retinal. Since PIP2 also contains one stearyl anchor, a saturated lipid, it is not clear how oxidation of only arachidonoyl anchor induces PIP2 solubilization. One possibility is that only a small fraction of distorted PIP2 undergoes additional cleavage from the stearyl anchor. This could result in a fraction of PIP2 molecules solubilizing to cytosol and induce calcium mobilization.

### Cell membranes are likely to facilitate retinal photoexcitation and cellular damage

As the location of retinal in the cellular environment could be crucial, the next objective was to examine the subcellular environment that can support the photochemical reactions of retinal. When a 20 µL solution of ATR in ethanol (50 mM) was exposed to blue LED light (5 W, 460–470 nm) for 30 minutes, a significant retinal degradation was observed (Fig. 7C). The retinal content in the solution was analyzed using high performance liquid chromatography (HPLC), before and after blue light exposure. HeLa cells expressing mCherry-PH sensor were incubated with 1 µL of this pre-blue light exposed retinal for 10 minutes. However, the addition of pre-blue light exposed retinal did not exhibit PIP2 distortion in cells. Exposure of these cells to blue light at 4.86 μW intensity also failed to exhibit a detectable mCherry-PH translocation, suggesting that (i) the mixture does not have sufficient retinal left and (ii) extracellularly generated retinal photoproducts are not able to disrupt cellular PIP2. Nevertheless, addition of fresh retinal (50 µM) followed by blue light exposure resulted in mCherry-PH translocation (Fig. 7C). Next, we examined if household white fluorescent light also induces retinal degradation, since both fluorescent and LED light contain a peak ~450 nm. Retinal degradation observed under white light was lower in ethanol (Fig. 7D). When retinal in hexane was exposed to white light, it showed even lower degradation. Interestingly, retinal in water exhibited over a 100 times faster degradation (within seconds) than in ethanol or hexane (within minutes) (Fig. 7D). These experiments collectively suggest that in cellular context, retinal must retain in a relatively non-aqueous and hydrophobic environment, not in the cytosol. These data also show that retinal undergoes efficient isomerization, in non-aqueous media upon photon absorption. PM lipid bilayer of cells is likely to provide an ideal environment for retinal to undergo continuous photoisomerization over degradation.

**Figure 8:**
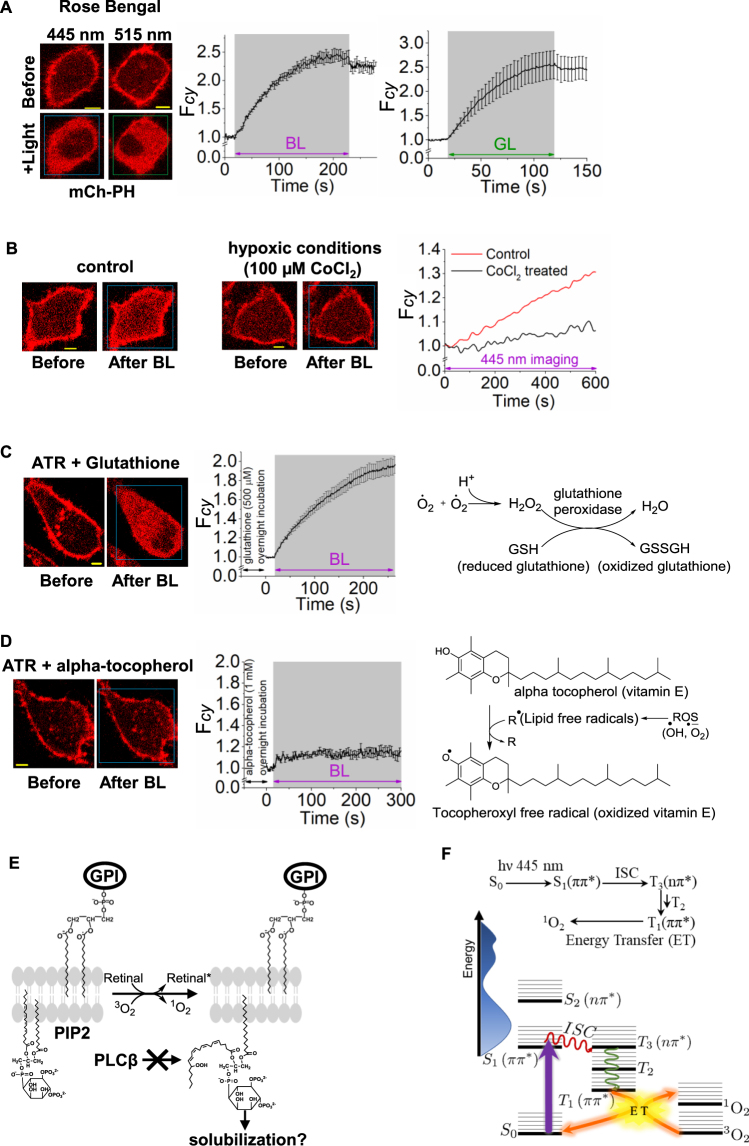
Comparison of PIP2 sensor dissociation from PM by retinal vs a known photosensitizer, rose bengal. (**A**) Images of HeLa cells expressing PIP2 sensor (mCherry-PH). (**A**) PIP2 sensor translocation was induced by rose bengal (50 µM), incubated with cells for 5 minutes, in the presence of blue (4.86 µW of 445 nm) and green (light (0.22 µW of 515 nm) respectively. Plot shows the cytosolic fluorescence of PIP2 sensor in HeLa cells upon exposing to light. (mean ± S.E.M., n = 6). (**B**) HeLa cells were incubated with CoCl_2_ (100 µM) for 24 h to expose cells to hypoxia. The control cells were kept in same conditions without CoCl_2_ treatment. Cells were incubated with ATR (50 µM) and 445 nm imaging for 10 minutes was performed. Cell in hypoxic condition did not exhibit detectable PIP2 sensor accumulation in cytosol while control cells showed a gradual PIP2 sensor accumulation from PM to cytosol. (**C**,**D**) Antioxidants were tested to examine if they prevent PIP2 sensor translocation induced by retinal and blue light. HeLa cells expressing PIP2 sensor were incubated with antioxidants, alpha-tocopherol (1 mM) and reduced-glutathione ethyl ester (500 µM) overnight. Prior to imaging experiments ATR (50 µM) was added and incubated for 5 minutes followed by exposure of blue light (4.86 µW of 445 nm) for 5 minutes. (**C**) Cells treated with reduced-glutathione ethyl ester exhibited PIP2 sensor translocation from PM to cytosol upon blue light exposure. Plot shows the dynamics of PIP2 sensor (mean ± S.E.M., n = 14 cells). Overview of the antioxidant mechanism exert by reduced glutathione *in vivo (right)*. (**D**) Cells treated with alpha-tocopherol showed a reduced rate and extent of PIP2 sensor translocation from PM to cytosol upon blue light exposure. Plot shows the dynamics of PIP2 sensor translocation (mean ± S.E.M., n = 6 cells). Note the reduction of PIP2 sensor accumulation in IMs of cells. *Right:* Overview of the antioxidant mechanism exert by alpha-tocopherol *in vivo*. (**E**) Proposed mechanism for blue light excited retinal induced PIP2 distortion process. (**F**) TD-DFT calculations (CAM-B3LYP/6–31++G**) of retinal’s energy states and the Jablonsky diagram shows strong absorption band due to the π → π* transition where triplet excited states are energetically and symmetrically matched to allow for efficient intersystem crossing and energy transfer to O_2_ which allows for singlet oxygen and ROS generation. Mean and S.E.M. are from 3 < independent experiments. (blue light (BL) = blue box and green light (GL) = green box). Scale = 5 µm.

Ability of retinal to harvest light and induce PIP2 distortion was examined to understand whether this process is governed by photosensitization of retinal. A known photosensitizer rose bengal (RB) absorbs green light (Fig. S9) and exhibits singlet oxygen generation^37^ and cell death^38^. HeLa cells expressing PIP2 sensor were incubated with RB (50 µM) and exposed to blue and green light respectively. Cells were imaged to observe whether RB too induces a similar PIP2 distortion. Both blue and green light exposure induced a significant PIP2 sensor translocation (Fig. 8A). To assess whether observed PIP2 distortion requires dissolved oxygen in cell culture media to produce singlet oxygen, HeLa cells were treated with CoCl_2_ for 12 hours to expose cells to hypoxia^39^. Upon exposure to BLE-retinal, compared to control normoxic cells, hypoxic cells exhibited an attenuated PIP2 sensor translocation (Fig. 8B). To validate that BLE-retinal induced PIP2 distortion was governed through free radical/ROS mechanisms, prior to blue light exposure, cells were incubated with antioxidants, glutathione ethyl ester and alpha-tocopherol respectively (Fig. 8C,D). Alpha-tocopherol is lipid soluble while glutathione is water soluble and cytosolic. Only cells incubated with alpha-tocopherol exhibited an attenuated PIP2 sensor translocation (Fig. 8C,D). This indicates that reactions of oxidative damage induced by BLE-retinal are likely taking place in lipid membranes (Fig. 8E).

To validate the ability of retinal to generate ROS via direct photoexcitation, spectroscopic calculations were performed with TD-DFT on the retinoids. The low-lying excited states of both 11CR and ATR are a series of strong singlet π → π* transitions and a dark singlet n → π* transition (Fig. 8F). The predicted absorption spectra for 11CR and ATR are in a reasonable agreement with the experimental spectra (Fig. S10A,B). For ATR, the strong absorption at 380 nm (S_1_) is the allowed HOMO to LUMO transition (π → π*), followed by a spectroscopically dark n → π* transition (S_2_). 11CR also has a similar spectral assignment. For both 11CR and ATR, S_1_ is nearly degenerate with a triplet state (^3^nπ*) and, by El Sayed’s rules, efficient intersystem crossing (ISC) should be observed where S_1_ (^1^ππ*) ISC to T_3_ (^3^nπ*). Interestingly, when examining the rotation around the 11-*cis* bond (Fig. S10C–E), S_1_ (^1^ππ*) and T_3_ (^3^nπ*) states cross with only a slight torsional twist about the 11-*cis* bond indicating that the two states are energetically degenerate to allow ISC. Once in the triplet manifold, the system should non-radiatively decay to the lowest triplet state (T_1_, ^3^ππ*) with sufficient energy (1.20 eV and 1.25 eV, relaxed T_1_ structure of 11CR and ATR) to transfer and promote oxygen to a cytotoxic singlet state (^3^O_2_(^3^Σ_g_^−^) → ^1^O_2_(^1^Δ_g_^−^) is 0.97 eV from experiment). The other retinoids studied are not expected to generate ROS because their absorptions occur in the UV (Fig. S3).

## Discussion

When absorption spectrum and λ_max_ of retinal (380 nm) are considered, blue light induced PIP2 distortion should be due to the ability of retinal to undergo photoexcitation because just addition of retinal alone or exposure to wavelengths longer than blue failed to respond. Multiple experiments conducted using inhibitors of G-proteins pathway suggest that, BLE-retinal mediated cellular responses are not due to retinal’s ability to interact with ligand binding GPCRs, further supporting the notion that BLE-retinal is directly responsible for its cellular effects. Lack of DAG formation observed in BLE-retinal mediated process further eliminates the possibility of PIP2 hydrolysis. Lack of sufficient spectral overlap in absorption spectra of retinol and retinoic acid with blue region is in agreement with their inability to induce cellular responses. In mouse mammary tumor cells, 10E, 12Z-CLA induced PIP2 hydrolysis and subsequent cell death was reported, and the activation of Gq-coupled GPCR(s) that results in PIP2 hydrolysis by activation of PLCβ has been proposed as the mechanism^40^. Nevertheless, in our experiments 10E, 12Z-CLA did not exhibit any effect on PIP2.

Acyl chains in PIP2 are crucial for its interactions with the PM. The difference in the acyl chain length in PIP2 have been shown to affect the localization of PIP2 on the PM^41^. If BLE-retinal modifies/changes properties of acyl chains, by oxidizing them, PIP2 is likely to lose the ability to stay bound to the PM. This can result in translocation of the modified PIP2, together with the bound mCherry-PH sensor to the cytosol. Alternatively, if the chemical modifications on PIP2 disrupt its interactions with mCherry-PH sensor as well as with PLCβ, irreversible translocation of PIP2 sensor to cytosol should be observed. However, using this argument, calcium responses induced by BLE-retinal cannot be explained. Therefore, it is likely that upon exposure to BLE-retinal, PIP2 becomes solubilized, at least to a certain extent, due to reduction of its PM affinity.

Increase in cytosolic calcium induced by BLE-retinal can be attributed to either (i) calcium influx from activated ion channels on PM or (ii) activation of IP3 receptors (IP3R) allowing ER to mobilize stored calcium or both. IP3R activation is likely to be induced by the IP3 moiety of solubilized PIP2 since it can still have the majority of interactions that IP3 possesses^33,42^. If solubilized PIP2 induces even a slight increase in cytosolic calcium, calcium activated ion channels on PM can further increase cytosolic calcium^43–45^. Lack of extracellular calcium resulted in reduction of calcium response compared to control cells, suggesting a partial involvement of the possibility (i) above. Inhibition of IP3 receptors by 2-APB which showed a reduction in calcium response compared to control cells by BLE-retinal suggests the possibility (ii). Collectively, these results suggest that calcium signaling induced by ATR and blue light is likely to be controlled by more than one mechanism.

PIP2 has been proposed to promote the interactions between PM and cytoskeleton, and cellular PIP2 levels are linked to bleb formation^46,47^. Therefore, observed extensive bleb formation in cells exposed to BLE-retinal can be a result of PIP2 activity reduction at PM. Additionally, BLE-excited retinal induced inclusion of propidium iodide into cells suggests activation of apoptosis-promoting pathways since excessive intracellular calcium results in cell death through apoptosis and necrosis^18^. Recent studies link ROS generation to non-inflammatory cell death termed as oxeiptosis through cellular ROS sensor and antioxidant factor KEAP1^48^. Although photodegradation products suggested to cause cytotoxicity in photoreceptor cells^6^, cells did not exhibit either PIP2 distortion or cell death on exposure to photoproducts of retinal. Our data show that household fluorescent white light (3 mW) induces extensive photo degradation of retinal in solution. However, at this intensity white light did not exhibit either PIP2 distortion or cell death, highlighting the blue light requirement for the process (Fig. 7A,B). Upon blue light exposure, accumulated lipophilic non-degradable lipofuscins have been shown to induce cytotoxicity in the retina^10–14^. Compared to the quantum yield of singlet oxygen ^1^O_2_ generation (*Φ*) by lipofuscin A2E, *all-trans* retinal exhibited a two times higher quantum yield (*Φ* = 0.2–0.3) in benzene^49^. While the quantum yield of ^1^O_2_ generation is vastly dependent on the solvent used, retinal usually exhibited higher quantum yield^50–55^ (Table S1). Our data show that retinal has enhanced lifetimes in relatively non-polar media and undergoes isomerization over degradation. Could retinal converted to A2E lipofuscins be responsible for its blue light induced cellular effects? Optimized conditions, such as two days in the dark in acidic medium were required for *in vitro* A2E synthesis using retinal and ethanolamine^56^. Therefore, it is unlikely that observed cellular effects within minutes are due to formation of A2E. Reactive singlet oxygen (^1^O_2_) forms due to excitation of molecular oxygen from its ground state to triplet state^57^. Singlet oxygen induces production of ROS such as superoxide, peroxide, and hydroxyl radicals^58,59^. The direct generation of ^1^O_2_ by absorption of a photon at 1270 nm (0.97 eV) is a weak transition due to the forbidden spin flip^60^. Thus, the proposed mechanism for generation of ^1^O_2_ involves a sensitizing chromophore which has a significant population of an excited triplet state with sufficient energy to excite ^3^O_2_ → ^1^O_2_ by electronic energy transfer^61^. Most chromophores are in a ground state singlet and gain triplet state population by ISC after initial photon absorption in the singlet manifold. ISC is usually spin forbidden. However, if there is a significant spin-orbit coupling via heavy atom effect or electronic transitions according to El Sayed rules, where ^1^ππ* state → ^3^nπ* state, ISC is observed^62^. Thus, we suggest that upon photoexcitation of ATR and 11CR to S_1_, retinal relaxes to triplet manifold by allowed ISC. Then the energy may be transferred to ^3^O_2_ generating ^1^O_2_. Collectively, both retinal-based experimental and computational data suggest that retinal dissolved in lipid bilayer is a major contributor for its blue light induced cellular effects.

Retinal is fat-soluble, and therefore transported through cytosol by carrier protein, Retinol/Retinal Binding Protein (RBP), while being protected against water^63^. When ethanolic solutions of retinal were dissolved in water, retinal was stable for a prolonged time in the dark (~1 week). However, exposure to light results in a rapid degradation. In contrast, retinal in relatively non-polar and non-aqueous media showed an enhanced photostability, suggesting that retinal prefers photoisomerization over degradation. Therefore, even if free retinal was present in cytosol, it is likely to degrade quickly. On the contrary, PM is an appropriate hydrophobic environment for retinal to be accumulated. Recently, transport of retinol in the hydrophobic core of serum amyloid A has been demonstrated^64^, further suggesting that PM core of cells can act as a reservoir of retinal. If photoexcited retinal can generate ^1^O_2_ in PM bilayer, it is more likely to interact with molecules in the PM. The ability of lipid soluble α-tocopherol to attenuate BLE-retinal induced PIP2 response also suggests that the resultant oxidative photodamage occurs within PM bilayer. Since PM and endoplasmic reticulum (ER), as well as mitochondria maintain direct contacts^65,66^, retinal has the potential to diffuse through the lumen of PM to ER, and to mitochondria. Although the presented data is limited to BLE-retinal induced signaling perturbation of PIP2, retinal’s photosensitizing ability in the cellular environment has the potential to elicit cell-wide oxidative damage to crucial signaling molecules and change the cellular fate.

## Materials and Methods

### Reagents

All *trans* retinal, retinol, retinoic acid, BAPTA, BAPTA-AM, conjugated (10*E*, 12*Z*) linoleic acid, propidium iodide (Cayman chemicals, Ann Arbor, USA), Beta-ionone (Acros Organics), 11*-cis* retinal (National eye institute, USA), YM254890 (Focus biomolecules), Gallein (TCI-America), Carbachol (Fisher Scientific), 2-APB, Pertussis toxin, SDF1α, alpha-tocopherol, glutathione ethyl ester (Sigma-Aldrich), Fluo4-AM (Molecular probes, Thermofisher), Rose bengal (Chem Impex) and Cobalt chloride (Alfa aesar) were purchased. All reagents were dissolved as per manufacturers’ recommendations unless otherwise noted. Stock solution of SDF1α was prepared in HBSS with 0.1% bovine serum albumin (Sigma-Aldrich). Propidium iodide was dissolved in water to make a working solution of 50 µg/mL. Rose bengal and Cobalt chloride were dissolved in water. Stock solutions of retinals (50 mM of 11CR or ATR) were prepared in Ethanol and dilutions were done appropriately using Ethanol and HBSS buffer. Concentrations of retinals were measured by UV-VIS spectrometer (UV-1800 spectrophotometer, Shimadzu corporation, Kyoto, Japan) or HPLC (CBM-20A, Shimadzu, USA) prior to live cell imaging experiments by plotting calibration curves with 11CR and ATR standards. In HPLC analysis of free retinal, a normal phase column (Waters Nova-Pak® Silica-60 Å, 4 µm, 3.9 × 150 mm) with an isocratic flow of 96:4 Hexane: Ethyl acetate at 0.5 mL/min flow rate was used. Deuterium lamp UV detector was used at 370 nm to identify retinals.

### Cell culture

HeLa cells (ATCC, Manassas, VA) were cultured in minimum essential medium (MEM) (CellGro) supplemented with 10% dialyzed fetal bovine serum (DFBS) (Atlanta Biologicals), in the presence of 1% penicillin−streptomycin (PS) in 35 mm/60 mm/100 mm tissue culture dishes at 37 °C in a 5% CO_2_ humidified incubator. At 70–80% confluency, adherent cells were detached using versene-EDTA (Lonza), centrifuged at 1000 × g for 3 minutes, and versene-EDTA was aspirated carefully without disturbing the cell pellet followed by addition of regular cell culture media at a cell density of 1 × 10^6^ cells/mL. For live cell imaging experiments, HeLa cells were cultured on 35 mm glass bottom dishes (*In Vitro* Scientific) with 1 × 10^5^/mL cell density. Types of cells and their respective cell culture media used in this study are as follows. RAW264.7 (RPMI/10% DFBS/1% PS), NIH3T3 (DMEM/10% BCS/ 1% PS), HEK293 (DMEM/10% DFBS/1% PS), MDA-MB-468, HCT116 and BT20 (DMEM/10% FBS/1% PS) and ARPE-19 (DMEM-F12 (50:50)/10% FBS/1% PS) (ATCC, Manassas, VA). Above mentioned cells were cultured using similar procedure as in HeLa cells.

#### Cell culture under hypoxic conditions

A procedure was followed as described previously^39^. Briefly, a solution of CoCl_2_ (25 mM) was prepared in water. HeLa cells were incubated with CoCl_2_ (100 µM) in the presence of regular cell culture media for 24 h at 37 °C in a 5% CO_2_ humidified incubator.

### *In vitro* transfection

HeLa, RAW264.7, NIH3t3, MDA-MB-468, HCT116, BT20, HEK293 and ARPE-19 cells were transfected using *in vitro* transfection reagent-PolyJet^®^ (Signagen) or lipofectamine2000^®^ reagent (invitrogen) according to manufacturer’s protocol with following recombinant DNA plasmids; mCherry-PH, YFP-PH, DBD-YFP, M3-muscarinic receptor-untagged, mCherry-γ9, bicistronic Melanopsin-GFP, CXCR-GFP, DenMark-mCherry, GPI-mCherry, GPI-GFP. Engineering of plasmids has been described previously^26,36,67–71^. Transfection was performed on cells seeded on glass bottom dishes. After 5 hours of incubation with the transfection reagent, the medium was replaced with 1 mL of fresh cell culture medium. Live cell imaging experiments were performed 12–24 hours after transfection.

### Live cell imaging, image analysis and data processing

Live cell imaging experiments were performed using a spinning-disk (Yokogawa CSU-X1, 5000 rpm) XD confocal TIRF imaging system composed of a Nikon Ti-R/B inverted microscope with a 60X, 1.4 NA oil objective and iXon ULTRA 897BVback-illuminated deep-cooled EMCCD camera. Photoactivation and spatio-temporal light exposure on cells in regions of interests (ROI) were performed using laser combiner with 40–100 mW 445, 488, 515, and 594 nm solid-state lasers equipped with Andor® FRAP-PA (fluorescence recovery after photobleaching and photoactivation) unit in real time, controlled by Andor iQ 3.1 software (Andor Technologies, Belfast, United Kingdom). Fluorescent sensors such as mCherry-PH, GPI-mCherry, mCherry-γ9, DenMark were imaged using 594 nm excitation−624 nm emission settings, YFP-PH and DBD-YFP were imaged using 515 nm excitation and 542 nm emission and GPI-GFP, Melanopsin-GFP, CXCR-GFP, Fluo4-AM were imaged using 488 nm excitation and 525 nm emission. Using a modulator, powers of solid state lasers were adjusted to 5 mW in above wavelengths for photoactivation studies where additional adjustments for laser power with 0.1–10% transmittance (0.22 µW–9.70 µW) were further achieved using Acousto-optic tunable filters (AOTF) to avoid photobleaching of fluorescent sensors. Continuous optical activation (OA) of cells expressing opsins was performed at every one second (1 Hz) using the 0.22 μW of 445 nm laser across the selected region and blue light exposure on cells without opsins was done with 4.86 μW of 445 nm laser. The laser power of light exposure was measured using a light meter (Ophir PD300-UV). Time-lapse images were analyzed using Andor iQ 3.1 software by acquiring the changes in mean pixel fluorescence intensity of the entire cell or in selected area/regions of interest (ROIs) of the cell. Briefly, background intensity of images was subtracted from the intensities of the ROIs assigned to the desired areas of cells (PM, IMs and cytosol), prior to intensity data collection from the time-lapse images. The intensity data from multiple cells were opened in Excel (Microsoft office®) and normalized to the base-line by dividing the whole data set with the average value of initial stable base-line. Data was processed further using Origin-pro data analysis software (OriginLab®).

### Light exposure induced PIP2 sensor translocation

Cells cultured on 35 mm glass bottom dishes were checked for PIP2 sensor (mCherry-PH) expression. After identification of cells expressing PIP2 sensor, the focal plane was locked to prevent the drifts in the imaging cross section using perfect focusing system (PFS). Next, cells were supplemented with 50 µM retinal (ATR or 11CR) for 5 minutes before light exposure on the cells. After retinal addition, cells were exposed only to 595 nm light to image mCherry fluorescence and all live cell imaging experiments were conducted in the dark. After capturing time-lapse images for mCherry at 1 Hz for 20–60 seconds, using the FRAP-PA module control of the assigned optical stimuli, whole cells or subcellular regions were exposed to light (445–594 nm laser light depending on the experiment) and time-lapse imaging was continued for additional 5–10 minutes. Additionally, 445 nm imaging was conducted to induce PIP2 solubilization in cells with hypoxic conditions (CoCl_2_ treated) and compared that with normoxic cells with same imaging parameters (445 nm excitation) without FRAP-PA.

### Cytosolic calcium measurements

Calcium imaging was performed with a cell permeable fluorescent calcium indicator, Fluo4-AM. Cells seeded on glass bottom dishes were washed twice with 1 mL of 1X HBSS (supplemented with calcium, pH 7.2) and incubated with Fluo4-AM (2.28 µM in 1X HBSS) for 30 minutes at room temperature. The glass bottom dish was washed with 1 mL of 1X HBSS three times before imaging experiments. The fluorescence intensity of Fluo4-AM was continuously imaged at 1 second intervals using 488 nm excitation −515 nm emission wavelength settings using confocal microscopy. Additional experiments were done in cells that were pre-incubated with Fluo4-AM (2.28 µM) followed by incubation of either 2-APB (5 µM for 15 minutes), BAPTA-AM (10 µM for 30 minutes), or BAPTA (5 µM for 5 minutes) in 1X calcium free HBSS buffer. The cells were then incubated with ATR (50 µM) for 5 minutes followed by continuous exposure of blue light for 5 minutes. The fluorescence intensity of Fluo4 was acquired. Fluorescence intensity obtained from regions of interest were normalized to initial values and data were further processed to obtain statistical data.

### Cell cytotoxicity imaging

Live cell imaging of HeLa cells was conducted with propidium iodide (PI) staining to assess cytotoxicity of cells upon exposing cells to either blue light only, ATR only, or exposing blue light in the presence of ATR. Incorporation of PI was monitored over time using time-lapse fluorescence imaging of cells. Briefly, cells were washed with 1X PBS and replaced with the cell culture medium. Then, 100 µL of PI (50 µg/mL) was added to the glass bottom dish with cells. The live cell imaging was started after selecting cells using differential interference contrast (DIC) microscopy. Blue light used here was 4.86 µW of 445 nm wavelength laser. DIC images and PI fluorescence were acquired over time. For PI imaging, 488 nm-excitation and 624 nm-emission filter settings were used. Final concentration of ATR was 50 µM used for experiments.

### HPLC analysis of retinal degradation

ATR (50 µM) solutions in different solvents (ethanol, hexane and water) were prepared using an ethanolic ATR stock solution (50 mM). ATR in ethanol was exposed to blue LED (5 W, 460–470 nm) up to 30 minutes. Similarly, ATR in ethanol and hexane were exposed to white fluorescent light (22 W) continuously up to 30 minutes. For retinal degradation studies in water, separate solutions were prepared and exposed to white light for time intervals from 1 s to 60 s in individual experiments. HPLC (CBM-20A, Shimadzu, USA) analysis of retinal degradation in each time interval was performed using a normal phase column (Waters Nova-Pak® Silica-60 Å, 4 µm, 3.9 × 150 mm) with an isocratic flow of 96:4 Hexane: Ethyl acetate at 0.5 mL/min flow rate was used. Deuterium lamp UV detector was used at 370 nm to identify percent retinal remaining in the solution. Percent retinal content in each sample was determined by calculating the percent area under each peak corresponding to type of retinals using Origin-pro data analysis software (OriginLab®).

### Light power measurements

Light power measurements were determined using a light meter, Ophir® PD-300UV (Ophir photonics, Israel) with the filter-in mode. StarLab® (Ophir photonics) software was used to acquire power measurements of lasers and white fluorescent light.

### Computational methods

Quantum chemical calculations were performed using time dependent density functional theory (TD-DFT) with the coulomb attenuating method (CAM-B3LYP) and a 6–31++G** basis set^72–74^. CAM-B3LYP and other long range corrected functionals have been shown to accurately determine spectroscopic properties and minimize the appearance of low lying spurious states^75^. All computations were performed using Gaussian09®^76^ in parallel with shared memory. Retinoids’ ground state and excited state energies in gas phase and in solution phase with polarized continuum model (PCM)^77^ were computed with heptane and water as the solvents.

## Electronic supplementary material

Supplementary Information

Movie-S1

Movie-S2

Movie-S3

Movie-S4

Movie-S5

Movie-S6
