# Supplementary material for: Blue light excited retinal intercepts cellular signaling
Source: Sci Rep. 2018 Jul 5;8:10207. doi: 10.1038/s41598-018-28254-8 (PMC6033873; doi:10.1038/s41598-018-28254-8)
Supplement: Supplementary file 1 — Supplementary Information [file 41598_2018_28254_MOESM1_ESM.pdf]

SUPPLEMENTARY INFORMATION

Blue light excited retinal intercepts cellular signaling

Kasun Ratnayake, John L. Payton, O. Harshana Lakmal and Ajith Karunarathne*

Department of Chemistry and Biochemistry, The University of Toledo, Toledo, OH 43606, USA

*Corresponding author: Ajith.karunarathne@utoledo.edu
To whom correspondence should be addressed: Dr. Ajith Karunarathne, Department of Chemistry and Biochemistry, The University of Toledo, 2801 West Bancroft Street, Toledo, OH-43606. Telephone: (419) 530-7880; FAX: (419) 530-4033; e-mail: Ajith.karunarathne@utoledo.edu

Supplementary figures and legends

Figure S1

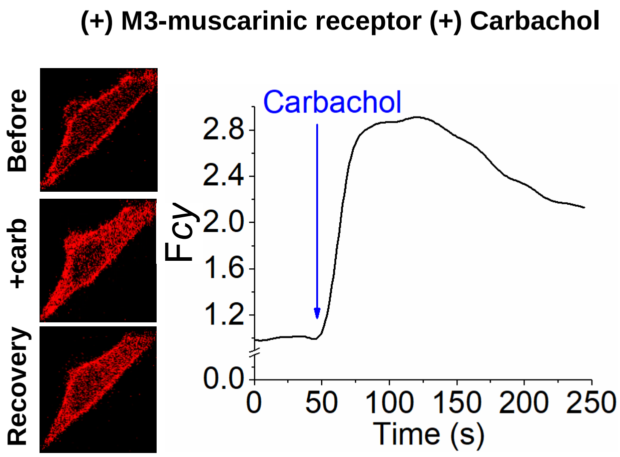

Figure S1: PIP2 hydrolysis by activation of M3-muscarinic receptors. Image of a HeLa cell expressing M3-muscarinic receptor (untagged), PIP2 sensor (mCherry-PH). M3-receptors were activated using carbachol (10 µM) and a robust PIP2 hydrolysis was observed. Within 2-3 minutes, the signaling was adapted shown by the recovery of PIP2 on PM. Plot shows the dynamics of PIP2. Scale= 5 µm.

Figure S2

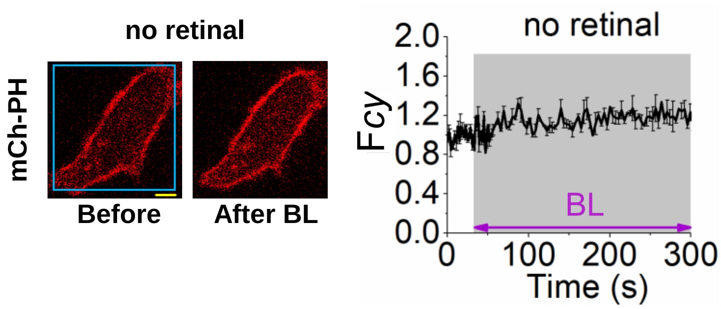

Figure S2. Blue light exposure alone on HeLa cells in the absence of retinal did not induce PIP2 sensor translocation. The plot shows the PIP2 sensor dynamics. (mean ± S.E.M, n = 3 cells). Mean and S.E.M are from three independent experiments. (blue light (BL) = blue box). Scale = 5 μm

Figure S3

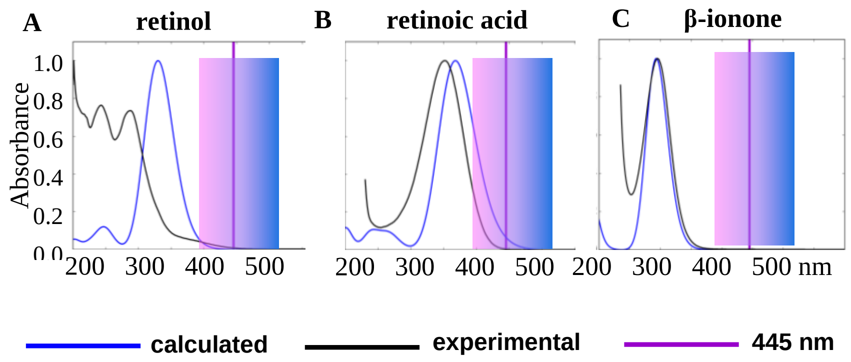

Figure S3: Experimental (black) and computed (blue) absorption spectra of retinoids, **A.** retinol, **B.** retinoic acid, **C.** β-ionone. Note the significant overlap of retinal spectra with blue light. The computed spectra are broadened by gaussian functions with a 0.25 eV half-width at half-maximum.

Figure S4

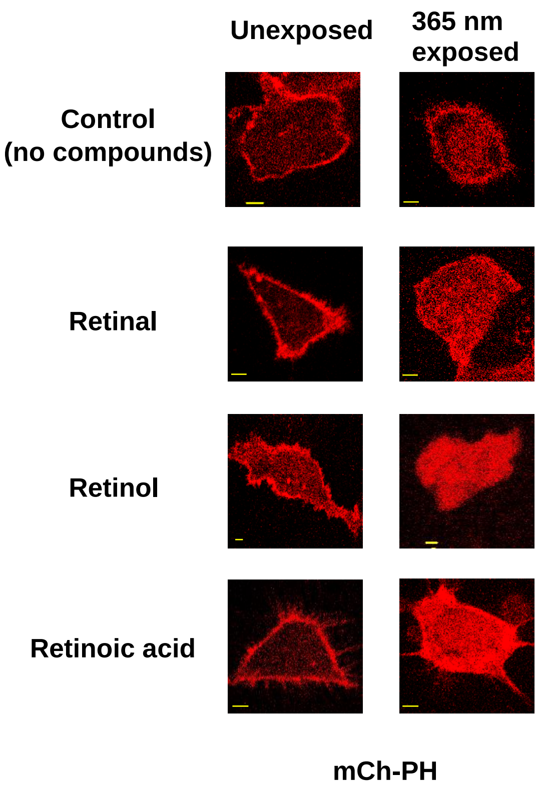

Figure S4. UV light exposure distorts PIP2 in cells in the presence of retinoids. Images of HeLa cells expressing PIP2 sensor, mCh-PH. UV light exposure (365 nm) on HeLa cells in the absence of retinoids did not induce PIP2 sensor translocation (control). Cells incubated with retinal, retinol and retinoic acid (250 µM) were exposed to UV light and exhibited a substantial PIP2 distortion. Note, that higher concentrations of retinoids were used to avoid photodegradation of compounds upon high energy UV light. Scale = 5 µm

Figure S5

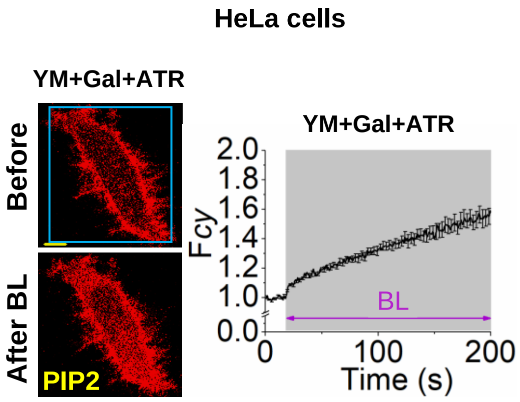

Figure S5: Photoexcited retinal induced PIP2 translocation in HeLa cells is not due to Gαq and Gβγ mediated PIP2 hydrolysis. HeLa cells expressing mCherry-PH were incubated with both Gβγ inhibitor (gallein, 10 µM, 30 min) and Gq inhibitor (YM254890, 1 µM, 5 min). Exposure to blue light excited retinal induced PIP2 sensor translocation in cells. Cells were exposed to 4.86 µW of 445 nm blue light which is indicated by the blue box. (mean ± S.E.M, n = 12 cells). Mean and S.E.M are from 3<independent experiments. (blue light (BL) = blue box). Scale= 5 µm.

Figure S6

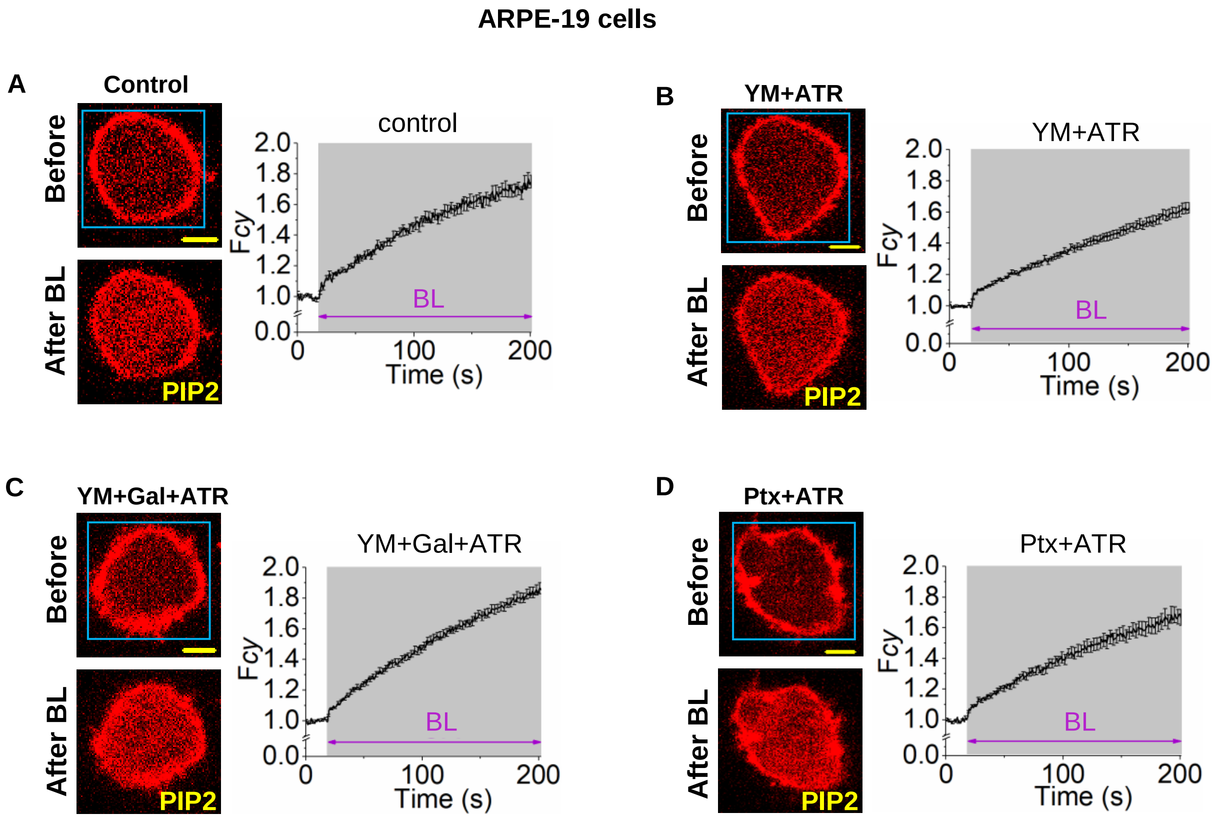

Figure S6: Photoexcited retinal induced PIP2 translocation exhibited by ARPE-19 cells is independent of GPCR-G protein pathway activation. ARPE-19 cells expressing mCherry-PH were incubated with **A.** no inhibitors, **B.** Gq inhibitor (YM254890, 1 µM, 5 min), **C.** both Gβγ inhibitor (gallein, 10 µM, 30 min) and Gq inhibitor (YM254890, 1 µM, 5 min), **D.** Gαi inhibitor (pertussis toxin=Ptx, 50 ng/mL, overnight incubation) followed by incubation with ATR (50 µM, 10 min) in dark. Upon exposure to blue light (4.86 µW of 445 nm-blue box), cells exhibited PIP2 sensor translocation from PM to cytosol. Plots show corresponding PIP2 sensor translocation. (mean ± S.E.M, n = 7-14 cells,). Mean and S.E.M are from 3<independent experiments. (blue light (BL) = blue box). Scale= 5 µm.

Figure S7

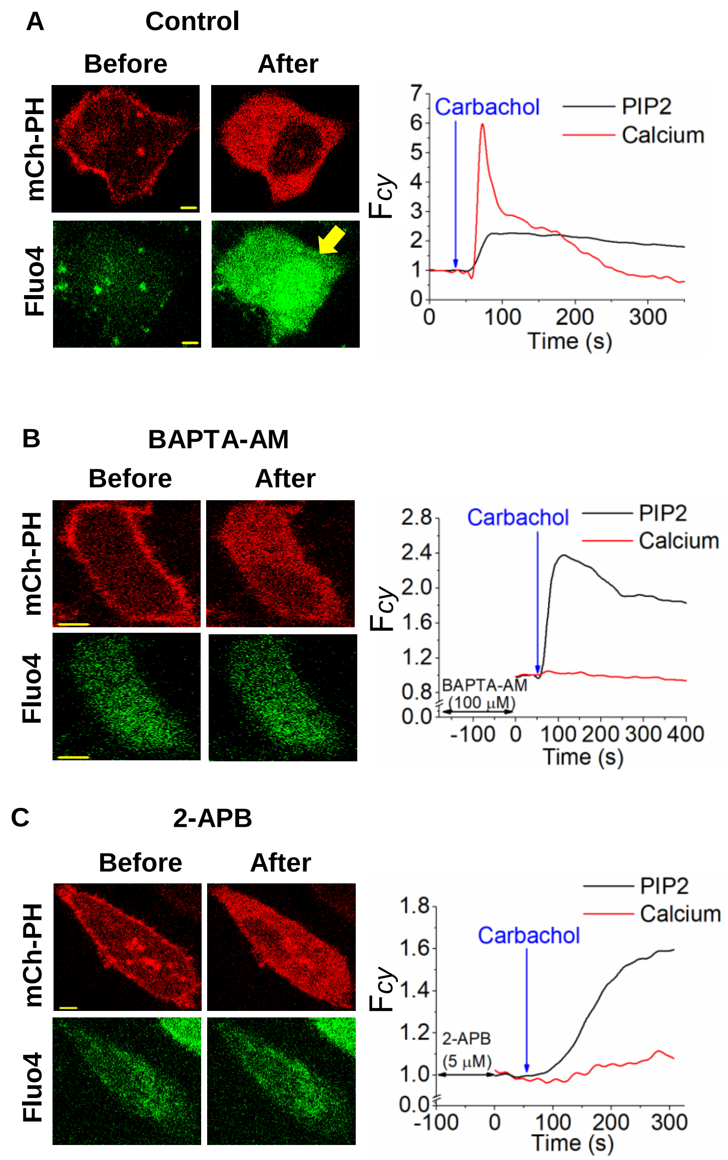

Figure S7: PIP2 hydrolysis and calcium signaling induced by activation of M3-muscarinic receptors in the presence of different experimental conditions. HeLa cells expressing M3-muscarinic receptor (untagged), PIP2 sensor (mCherry-PH) and calcium sensor (Fluo4) **A.** M3-receptors were activated using carbachol (10 µM) to induce PIP2 hydrolysis and calcium signaling. Plot shows the dynamics of PIP2 and calcium signaling shown in A. **B.** Cells were incubated with BAPTA-AM (100 µM, 20 minutes) to chelate intracellular calcium while addition of carbachol (10 µM) to cells did not induce neither PIP2 nor calcium signaling. Plot shows the dynamics of PIP2 and calcium signaling shown in B. **C.** Incubation of cells with an inhibitor of IP3 receptor, 2-APB (5 µM, 10 minutes), followed by addition of carbachol (10 µM) resulted only PIP2 hydrolysis but not calcium responses. Plot shows the dynamics of PIP2 and calcium signaling shown in C. Scale= 5 µm.

Figure S8

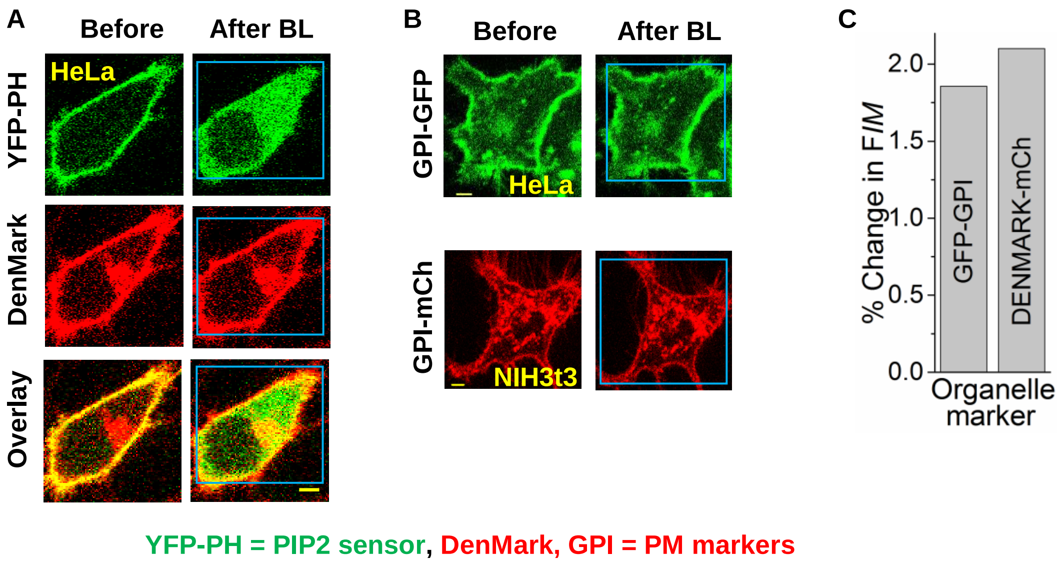

Figure S8. Retinal and blue light induced PIP2 sensor falling off in cells is not an artifact of fluorescent biosensor. A. A HeLa cell expressing dendritic marker (DenMark-mCh) and YFP-PH was exposed to blue light (4.86 µW of 445 nm) in the presence of ATR (50 µM). Only YFP-PH is translocated into the cytosol from PM but not DenMark-mCherry. **B.** ATR (100 µM) was added to HeLa and NIH3t3 cells expressing GFP-GPI and mCherry-GPI, respectively, followed by blue light (4.86 µW of 445 nm) exposure on cells where no accumulation of fluorescence was observed in exposed cells. **C.** %Change in IM fluorescence of organelle markers, GFP-GPI and DenMark upon ATR and blue light illumination. (blue light (BL) = blue box). Scale = 5 µm

Figure S9

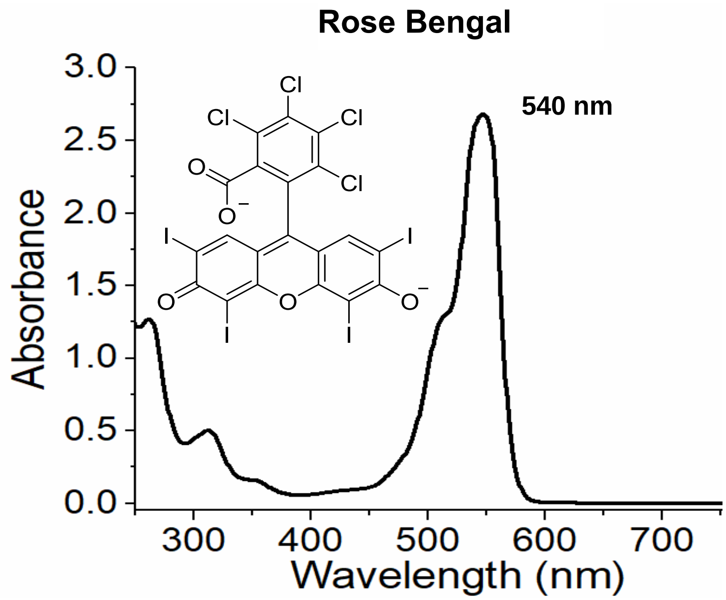

Figure S9. Structure of rose bengal (RB) and its UV-VIS spectrum in aqueous solution (25 µM).

Figure S10

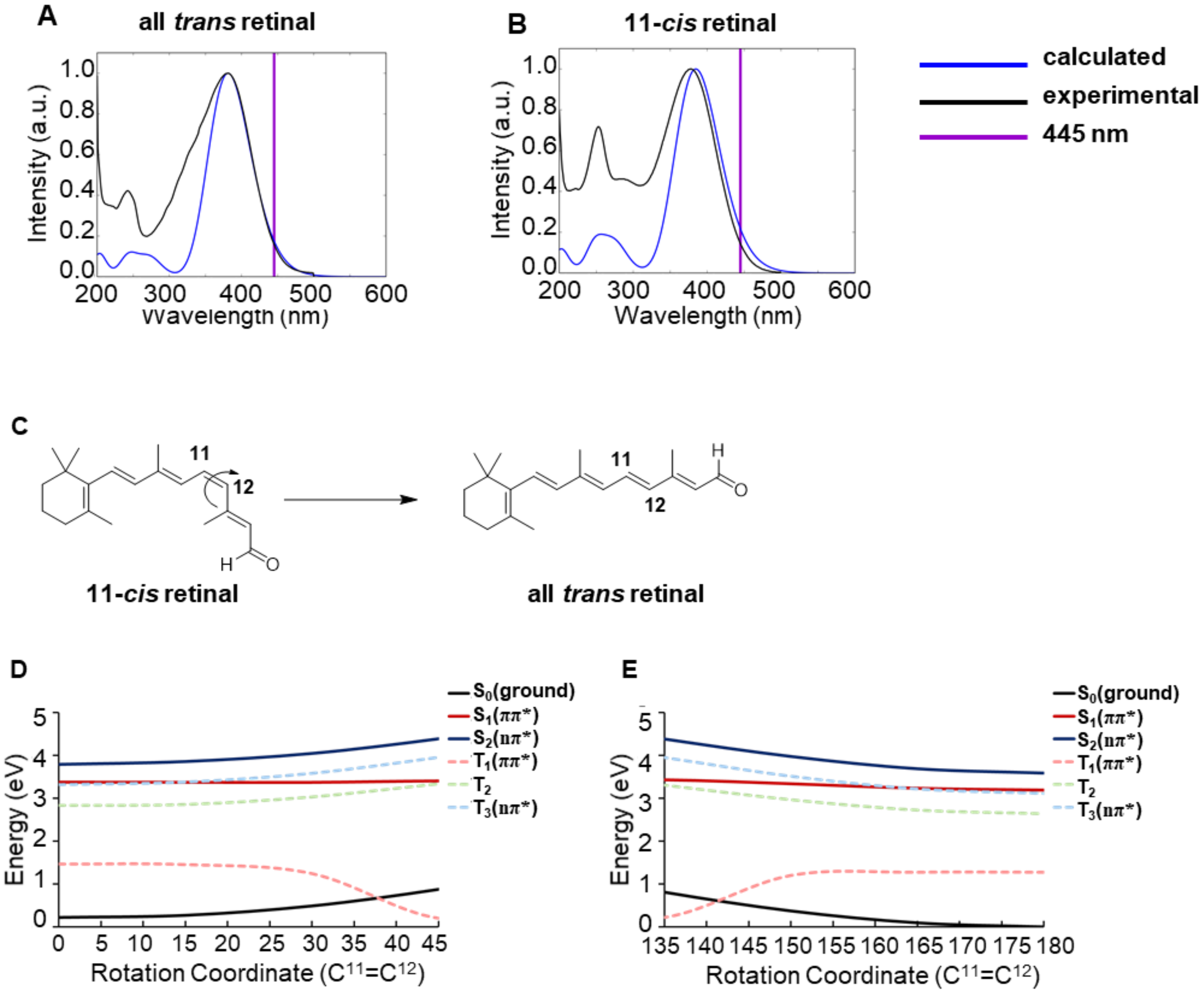

Figure. S10. TD-DFT calculations (CAM-B3LYP/6-31++G**) of absorption spectra of retinals. **A and B.** (A: all-*trans*-retinal (ATR), B: 11-*cis*-retinal (11CR). The computed (blue) and experimental (black) UV-VIS absorption spectra are shown with 445 nm laser emission line (violet line). The computed spectra are broadened by gaussian functions with a 0.25 eV half-width at half-maximum. The TD-CAM-B3LYP/6-31++G** potential energy surfaces are plotted about the torsional angle shown in **C** for 11CR (**D**) and ATR (**E**). Potential energy surfaces were plotted as a splined function of the data points at 15° increments.

Table-S1

**Table-S1, Retinals are likely to be more phototoxic than lipofuscin: Comparisom of quantum Yields (Φ) of $^1O_2$ generation[1,2]**		
**Molecule**	Φ (λ_{max}) nm	***Solvent***
lipofuscin	0.05 (440)	C_6H_6
lipofuscin	0.08 (355)	C_6H_6
All trans retinal	0.20 (337)	C_6H_6
All trans retinal	0.55	CCl_4
11-cis retinal	0.55	CCl_4

1. Krasnovsky, A. A., Jr. & Kagan, V. E. Photosensitization and quenching of singlet oxygen by pigments and lipids of photoreceptor cells of the retina. *FEBS Lett* **108**, 152-154 (1979).
2. Chattopadhyay, S. K., Kumar, C. V. & Das, P. K. Laser flash photolytic determination of triplet yields via singlet oxygen generation. *Journal of Photochemistry* **24**, 1-9, doi:https://doi.org/10.1016/0047-2670(84)80001-5 (1984).

Supplementary movie legends

Supplementary movie 1

Exposure of blue light (4.86 µW of 445 nm) on HeLa cell expressing PIP2 sensor (mCherry-PH) in the presence of all *trans* retinal (50 µM) (light exposure=white box). Scale = 5 µm

Supplementary movie 2

Melanopsin was activated using blue light (0.22 µW of 445 nm) in a HeLa cell expressing PIP2 sensor (mCherry-PH) in the presence of all *trans* retinal (50 µM). The reversal of the PIP2 sensor translocation can also be observed (light exposure=white box). Scale = 5 µm

Supplementary movie 3

M3-muscarinic receptor was activated using carbachol (10 µM) in a HeLa cell expressing PIP2 sensor (YFP-PH). The reversal of the PIP2 sensor translocation can also be observed. Scale = 5 µm

Supplementary movie 4

Exposure of blue light (4.86 µW of 445 nm) on a selected HeLa cell (left, yellow box) expressing PIP2 sensor (mCherry-PH) in the presence of all *trans* retinal (50 µM). Control cell is shown on the right without blue light exposure (light exposure=white box). Scale = 5 µm

Supplementary movie 5

Exposure of blue light (4.86 µW of 445 nm) on a HeLa cell incubated with calcium sensor (Fluo4-AM) in the presence of all *trans* retinal (50 µM). Note, the increase in cytosolic calcium upon light exposure (white box). Scale = 5 µm

Supplementary movie 6

Exposure of blue light (4.86 µW of 445 nm) on a selected HeLa cell in the presence of all *trans* retinal (50 µM). Prolonged exposure of blue light (~45 min) on HeLa cell (middle) show bleb formation and morphological change in plasma membrane (light exposure=white box). Scale = 5 µm
